# Do better executive functions buffer the effect of current parental depression on adolescent depressive symptoms?

**DOI:** 10.1016/j.jad.2016.03.049

**Published:** 2016-07-15

**Authors:** Shiri Davidovich, Stephan Collishaw, Ajay K. Thapar, Gordon Harold, Anita Thapar, Frances Rice

**Affiliations:** a Department of Clinical, Educational and Health Psychology, University College London, United Kingdom; b Child and Adolescent Psychiatry Section, Institute of Psychological Medicine and Clinical Neurosciences, School of Medicine, Cardiff University and MRC Centre for Neuropsychiatric Genetics and Genomics, Cardiff University, United Kingdom; c Rudd Centre for Adoption Research and Practice, School of Psychology, University of Sussex, United Kingdom; d International Centre for Research in Human Development, Tomsk State University, United Kingdom

**Keywords:** Depression, Adolescence, Executive functions, High-risk, Cognitive, Resilience

## Abstract

**Background:**

Offspring of parents with a history of major depressive disorder (MDD) and especially those exposed to a current episode of parental depression have been found to be at increased risk for developing depression themselves. Exposure to a current parental depressive episode also reduces the efficacy of interventions in high risk or depressed adolescents. This highlights the need to identify protective factors for adolescents exposed to a current parental depressive episode. Executive functions serve as an important cognitive resource, involved in the ability to regulate mood and thoughts and cope with stressful events. This study examined the buffering role of two components of executive functioning, inhibitory control and mental flexibility, in the association between a current parental episode of MDD and adolescent depressive symptoms.

**Methods:**

A high-risk sample of 288 adolescent offspring of parents with recurrent major depressive disorder completed an Affective Go/No Go and a Verbal Fluency task. Parents and adolescents underwent psychiatric interviews.

**Results:**

In the presence of a current parental depressive episode in the parent, adolescents with better inhibitory control and mental flexibility had fewer depressive symptoms after controlling for age, gender and IQ.

**Limitations:**

Participants were the offspring of depressed parents and it is not clear whether the protective effects of executive functioning observed here would generalise to other populations.

**Conclusions:**

Executive functions may protect against adolescent depression in the presence of a parental depressive episode. It may be beneficial to target executive functions in preventive programs for individuals at high-risk for depression.

## Highlights

• Do executive functions (EF) protect against depression in high-risk youth?
• Participants were parents with recurrent MDD and their adolescent offspring.
• Adolescents exposed to a current parental episode had more depressive symptoms.
• High EF served as a buffer for adolescents exposed to a current parental episode.
• Targeting EF in interventions for high risk groups may be beneficial.

## 1 Introduction

Parental depression has been identified as a major risk factor for depression in childhood and adolescence with children of depressed parents three to four times more likely to develop depression than offspring of non-depressed parents (Garber, 2006, Rice and Rawal, 2010, Weissman et al., 1997). Despite the strong association found between parental depression and offspring depression, there is heterogeneity in outcomes for children of depressed parents which is partly attributable to clinical features of parental depression. Evidence suggests that exposure to a current parental depressive episode is an important feature of parental depression that increases risk for offspring. For instance, children of parents with a history of recurrent depression whose parents have had a recent episode of major depressive disorder (MDD) show elevated rates of psychiatric disorder and depressive symptoms (Mars et al., 2012). Exposure to a recent episode of depressive disorder in a parent has also been shown to moderate the efficacy of treatment and prevention programs (Beardslee et al., 2013, Brent et al., 1998, Garber et al., 2009). Thus, exposure to a current parental episode may serve as a particularly salient risk factor among those at familial risk of depression. This evidence, taken together with the long-term adverse consequences of depression in childhood and adolescence (Dunn and Goodyer, 2006, Fergusson et al., 2007, Rice et al., 2007, Rutter et al., 2006) emphasizes the need to identify protective factors for high-risk offspring and especially for those currently exposed to a depressive episode in the parent.

Several lines of evidence suggest that executive functions may confer protection against depression for the adolescent offspring of depressed parents. First, cognitive models of depression suggest that the mood regulation difficulties that characterise currently depressed individuals (De Lissnyder et al., 2010, Harmer et al., 2009, Roiser et al., 2011) may arise from difficulties with executive functions such as inhibition and mental flexibility. Inhibitory control involves controlling attention, thoughts and behaviours in order to override an automatic or dominant response and mental flexibility allows switching between different mental sets or perspectives in order to adjust to changing or novel circumstances (Davidson et al., 2006, Diamond, 2013, Miyake et al., 2000). It is plausible that the ability to inhibit thoughts and flexibly switch between thoughts and perspectives can protect against being “captured” by negative thoughts or low mood. Thus, both inhibitory control and mental flexibility have been associated with more effective emotional regulation strategies including lower levels of rumination – a cognitive style which perpetuates negative affect (Gotlib and Joormann, 2010, Joormann and Quinn, 2014) and higher levels of reappraisal – an effective mood repair strategy (McRae et al., 2012, Ochsner and Gross, 2008). Studies conducted with depressed adults have indicated particular impairments in inhibitory control and mental flexibility when processing emotional information (Deveney and Deldin, 2006, Gotlib and Joormann, 2010, Murphy et al., 2012). Although fewer studies have examined if these impairments occur in depressed children and adolescents, several lines of evidence suggest these deficits are also present in depressed children and adolescents (Kyte et al., 2005, Ladouceur et al., 2005, Wagner et al., 2014). Moreover, preliminary evidence suggests that difficulties in executive functioning, such as impairments in inhibition on emotional tasks, may precede and increase risk for depression (Joormann et al., 2007, Kilford et al., 2014).

A second line of evidence that suggests executive functioning may protect against depression is that efficacious interventions for treating and preventing adolescent depression such as Cognitive Behavioural Therapy (CBT) (Stice et al., 2009, Weisz et al., 2006) involve training in evaluating and challenging thoughts and introducing alternatives (Forkmann et al., 2014, Friedberg and McClure, 2002). These mood regulation skills involve elements of executive functioning, for instance in inhibiting negative thoughts (i.e. inhibition) and thinking about an issue from a different perspective to introduce alternative thoughts (i.e. mental flexibility). In a similar way, when healthy individuals are instructed to look at an emotionally salient event from different perspectives this has significant impact on emotional regulation and reactivity as measured by self-report and physiological measures (Kross et al., 2005, Schartau et al., 2009, Southwick et al., 2005).

A third source of evidence comes from findings which suggest that executive functions may serve as an important cognitive resource that protects individuals at familial risk of psychiatric disorders (Johnson, 2012). Johnson (2012) suggests that better executive functions may help those at genetic risk for developmental disorders by allowing compensatory brain systems to be recruited during cognitive operations. Thus, it is plausible that executive functions may also serve as a protective factor in those at increased familial risk of depression.

Although previous research has shown that currently depressed individuals are characterized by executive functioning impairments and that these impairments may increase risk for depression, past studies have not directly examined whether better executive functions serve as a protective factor for those at increased familial risk of depression. In order to address this gap in the literature, we aimed to examine the protective effect of executive functioning (inhibitory control and mental flexibility) in a high risk sample of adolescent offspring of parents with a history of recurrent MDD. Our primary research question was whether inhibitory control and mental flexibility protected against the predicted risk effect of a current parental depressive episode on adolescent depressive symptoms. On the basis of evidence involving inhibitory control and mental flexibility with dysregulation of thoughts and emotions we also examined whether the proposed moderating effect of executive functions was present for both cognitive-emotional depressive symptoms (e.g. low mood/irritability and concentration difficulties) and for vegetative-somatic depressive symptoms (e.g. change in appetite or weight and psychomotor retardation/agitation). We hypothesized that for adolescents exposed to a current parental episode, the buffering effect of executive functions would be more consistently present for cognitive-emotional depressive symptoms than for vegetative-somatic depressive symptoms given that the former indicate dysregulation of thoughts and emotion and therefore might be more attenuated in those with better executive functions.

## 2 Methods

### 2.1 Participants

Participants were a part of a longitudinal study of parents with recurrent major depression and their adolescent offspring (aged 9-17), the Early Prediction of Adolescent Depression (EPAD) study. Parents were recruited from general practices in south Wales (78%), from a previous community study of recurrent unipolar depression (19%) and from advertisements in primary care health centres (3%). Following initial screening over the phone, parents underwent a psychiatric interview to confirm a history of recurrent unipolar depression. Families were excluded if the index parent met criteria for a bipolar disorder, mania/hypomania or psychotic disorder at the time of the interview; the child wasn’t living at home or had an IQ lower than 50. If more than one child in the household was eligible to participate in the study, the youngest child was selected in order to prevent selection bias. The EPAD study was undertaken with approval of the Multi- Centre Research Ethics Committee for Wales. Assessments were administered in families’ homes. The present study utilizes data collected at the second assessment of the study because adolescents completed a battery of cognitive tests including measures of executive functioning at this assessment.

At the second assessment of the study 288 parents and 275 adolescents completed research psychiatric interviews, the Child and Adolescent Psychiatric Assessment (CAPA), for assessing psychopathology in the offspring. Among 288 adolescents for whom a psychopathology assessment was completed (by either the parent and offspring or by the parent only), data on performance on the Verbal Fluency (VF) task was available for 264 (91.6%) adolescents and data on performance on the Affective Go/No-Go Task (AGN) was available for 187 (64.9%). Fig. S1 outlines participation rates and reasons for non-completion. There were no systematic differences between adolescents who completed the Verbal Fluency task and the AGN and those who did not in terms of gender [VF: χ^2^(1)=1.52,p=.22; AGN: χ^2^(1)=.10,p=.75 ], age [VF: t(286)=1.40, p=.16; AGN: t(286)=.89, p=.37] and depressive symptoms [VF: t(282)=.96, p=.34; AGN: t(282)=−.07, p=.95]. However, participants who completed the VF and AGN had higher IQ scores than those who did not [VF: t(328)=−4.54, p<.001; AGN: t(328)=−2.83, p<.01]. IQ was therefore included as a covariate in all analyses that follow. As detailed in Table 1, the mean number of CAPA defined adolescent depressive symptoms was 1.93. Additionally, 6% of adolescents in this sample met DSM-IV criteria for major depressive disorder (MDD). These rates are substantially higher than those found in community studies where similarly stringent criteria are used (Costello et al., 2003, Green et al., 2005, Silberg et al., 1999).

### 2.2 Measures

#### 2.2.1 Parent Psychiatric Assessment

Depressive symptoms and disorder in the parent were assessed using the Schedules for Clinical Assessment in Neuropsychiatry(SCAN; Wing et al., 1990).The primary exposure variable was the presence/absence of a DSM-IV episode of MDD in the parent. However, we also assessed past parental severity (the presence of a previous severe episode), parental age of onset and child exposure to previous parent depressive episodes (the number of previous parental depressive episodes the child was exposed to). These variables were included as covariates in analyses that follow. The SCAN is a psychiatric interview which provides a detailed assessment of adult psychopathology. A life history calendar approach was used to compile a timeline of the affected parent’s previous episodes and to define age of onset (Caspi et al., 1996, Freedman et al., 1988). Based on the SCAN and the timeline data, the presence of a parental current episode of major depressive disorder (MDD), the presence of a previous severe episode, parental age of onset and child exposure to previous parent depressive episodes were defined according to the following criteria: Parent current depressive disorder was defined as whether an episode of DSM-IV defined MDD had occurred in the previous month (yes; no); Parent age of onset was dichotomized to ≤20 versus 21 years or older in accordance with previous research (Weissman et al., 1984); A previous parental episode was considered severe if it involved severe functional impairment (a score of GAF≤30 on the Global Assessment of Functioning scale in DSM–IV; American Psychiatric Association, 1994) or hospitalization (Hammen and Brennan, 2003, Mars et al., 2012); using a life history calendar approach, a count of the number of parental episodes the child was exposed to from birth until the year prior to the current assessment was calculated.

#### 2.2.2 Adolescent Psychiatric Assessment

Depressive symptoms in the adolescent were assessed using the Child and Adolescent Psychiatric Assessment (CAPA; Angold and Costello, 2000). The CAPA is a semi-structured interview which assesses psychopathology over the previous 3 months. Interviews were conducted separately both with the parent and the adolescent. Adolescent total number of depressive symptoms was the primary outcome variable (range 0–9). Cognitive-emotional and vegetative-somatic symptoms were also considered separately as secondary outcome variables using definitions based on previous research (Cavanaugh, 1984, Cook et al., 2010, Osman et al., 2004). Number of cognitive-emotional symptoms included the symptoms of irritable or low mood, loss of interest, feelings of worthlessness or guilt, inefficient thinking/indecisiveness, suicidal thoughts/plans/behaviour (range 0–5). Number of vegetative-somatic symptoms included the symptoms of change in appetite, sleep disturbance, loss of energy, psychomotor agitation or retardation (range 0–4). The three outcome measures of symptom counts consisted of a combined report of the parent and the adolescent such that a symptom was considered present if it was reported by either the child or the parent, as supported by common clinical practice (Angold et al., 1995, Costello et al., 2003). Sensitivity analyses additionally examined adolescent-rated depressive symptoms as the outcome variable. Attention Deficit Hyperactivity Disorder (ADHD) was also assessed using the CAPA and analyses were re-run excluding individuals meeting DSM-IV criteria for any of the three subtypes of ADHD: the combined type, the predominantly inattentive type and the predominantly hyperactive-impulsive type.

#### 2.2.3 IQ

The 10 subscales from the Wechsler Intelligence Scale for Children- Fourth edition (WISC-IV; Wechsler, 2003) were used to measure full-scale IQ.

#### 2.2.4 Executive functioning

The Verbal Fluency task (VF; Benton, 1968) was administered to assess mental flexibility (Diamond, 2013, Suchy, 2009). Participants are required to generate as many words as possible beginning with the letters F, A, S with 1 minute given for each letter. Participants were instructed that proper nouns (e.g. France), repetitions and more than one word of the same origin (e.g. act, acting) were not acceptable and they were given examples of each. The task involves the ability to devise different strategies for coming up with as many words as possible and to generate categories from which to extract words (Fossati et al., 2003). Outcome measures were: 1) Total number of correct responses; 2) number of disallowed responses i.e. words that violated the rules of the task; 3) number of repetitions (Regard et al., 1982, Turner, 1999).

The Affective go/no go task (AGN; www.camcog.com; Murphy et al., 1999) was used to measure inhibitory control and set-shifting. A series of words is rapidly presented in the centre of the screen. Words can be positive or negative. Participants are given a target valence and asked to press the press pad when they see a word that matches this valence, while withholding response to words of the other valence (distractors). There are 2 practice blocks and 8 test blocks of 18 words each. The target word changes during the task, so that half of the blocks are shift blocks. The task measures inhibitory control and set-shifting ability in the context of processing emotional information. A number of outcomes measures were initially examined: 1) Commission errors (the number of responses to distractor stimuli i.e. false alarms) for all trials and for shift blocks only. 2) Omission errors (the number of missed responses to targets) for all trials and for shift blocks only. 3) Shifting costs (calculated as the difference between the mean reaction time in non-shift blocks and the mean reaction time in shift blocks). Larger differences represent difficulties in set shifting ability (Karbach and Kray, 2009).

### 2.3 Statistical analysis

Exploratory Factor Analysis (EFA) on all outcome measures from the Verbal Fluency and Affective Go No Go Tasks was used to identify measures assessing inhibitory control and mental flexibility which are unique components of executive functioning (Diamond, 2013, Suchy, 2009). This derived one measure of inhibitory control errors (number of commission errors on the AGN) and two measures of mental flexibility (number of correct responses on the Verbal Fluency task and AGN shifting costs).

In addressing the main hypotheses, we first examined the main effect of current parental depression on child executive functioning variables using linear regression while controlling for the additional parental depression variables (the presence of a previous severe episode, age of onset and child exposure to previous parent depressive episodes). Next, we tested the main effect of current parental depression on adolescent depressive symptoms and assessed whether any observed association remained when controlling for the covariates of the presence of a previous severe episode in the parent, parent age of onset and child exposure to previous parent depressive episodes. We entered current parental depression and the three covariates mentioned above in separate steps of the regression in order to examine whether these covariates had additional contribution to the regression models above current parental depression.

We next examined executive functioning variables as moderators of the association between current parental depression and offspring depressive symptoms using linear regression. Analyses were conducted separately for each of the three measures of executive functioning identified by the EFA. Independent and control variables were standardized (Cohen et al., 2003). Covariates identified as significantly associated with EF measures or with offspring depressive symptoms were retained in the test of moderation. In the first step of each regression, adolescent age and gender were entered as covariates. Next, full scale IQ was entered as a covariate. In the third step, the main effects of current parental depression (currently affected yes (1); no (0)) and the measure of executive functioning were included. In the fourth step the interaction term between current parental depression and executive functioning was entered. Significant interactions were illustrated by plotting counts of depressive symptoms for adolescents with high (top tertile) and low (bottom tertile) performance on executive functioning measures by current parental depression. In order to rule out the possibility that the observed pattern of results was attributable to biased rating of adolescent depressive symptoms by currently depressed parents, analyses were repeated with adolescent-rated depressive symptoms as the outcome variable. To rule out the possibility that ADHD served as a confounder as it has been associated with both executive functioning impairment and higher depressive symptoms (Humphreys et al., 2013, Willcutt et al., 2005), we repeated the analysis excluding adolescents meeting DSM-IV diagnostic criteria for ADHD (n=16). Additionally, we repeated the analyses while controlling for the three additional parental depression variables examined earlier (the presence of a previous severe episode, age of onset and child exposure to previous parent depressive episodes). Finally, we examined whether the buffering effect of executive functioning was present for both cognitive-emotional symptoms and for vegetative-somatic symptoms by conducting linear regression separately for each symptom group.

## 3 Results

### 3.1 Deriving inhibitory control and mental flexibility components of offspring executive functioning

An exploratory factor analysis using principal component extraction and direct Oblimin rotation was performed on adolescent executive functioning measures (Table 2). This derived four factors, three that assessed aspects of executive functioning and one that assessed attention. High factor loadings of individual measures as well as distributive properties and theoretical understanding of the construction of executive functioning (Suchy, 2009) were the criteria used to select measures tapping unique aspects of executive functioning for analysis. This resulted in one measure of inhibitory control errors i.e. failure to inhibit an inappropriate behavioural response to a distractor (number of commission errors on the AGN) and two measures tapping separate aspects of mental flexibility i.e. mental generativity and shifting costs. Mental generativity was assessed by number of correct responses on the Verbal Fluency task which is suggested to represent the ability to generate and create ideas and responses to problems (Hendrawan et al., 2012, Suchy, 2009). Although both VF repetitions and VF correct responses had loadings higher than .4 on the mental generativity factor, we chose to focus on VF correct responses over VF repetitions since repetitions rarely occurred (Table 2) and the measure of VF correct responses has received more support in previous studies as a measure of mental generativity (Hendrawan et al., 2012, Suchy, 2009). AGN shifting costs was assessed as the difference in reaction times between shift blocks and non-shift blocks with larger differences representing greater difficulty in shifting (Karbach and Kray, 2009). Results of the exploratory factor analysis suggested that mental generativity and shifting costs constitute two separate aspects of mental flexibility (Table 2).

### 3.2 Preliminary analyses

Correlations between all study variables and descriptive statistics are presented in Table 1. Measures of executive functioning were not significantly associated with adolescent depressive symptoms nor with indicators of current parental depression, the presence of a previous severe episode in the parent, parental age of onset or child exposure to previous parent depressive episodes (Table 1). Linear regression analyses were conducted to examine the main effect of parental current depression on adolescent executive functioning and adolescent depressive symptoms while controlling for age, gender and the additional parental depression variables. As presented on Table 3, after controlling for age, gender and additional parental depression variables, parental current depression was not significantly associated with adolescent executive functioning (Parental current depression: AGN inhibitory control errors β=−.03, p=.66; VF mental generativity β=−.10, p=.12; AGN shifting costs β=.04, p=.65). However, as predicted, current parental depression was significantly associated with adolescent depressive symptoms after controlling for age and gender [β=.20, p<.01]. This effect was present when controlling for the covariates of the presence of a previous severe episode in the parent, age of onset in the parent and child exposure to previous parent depressive episodes. The covariates of parent previous severe episode, parent age of onset and child exposure to previous parent depressive episodes were not found to be significantly associated with offspring executive functions [parent previous severe episode: AGN inhibitory control errors β=.01, p=.86, VF mental generativity β=.06, p=.40, AGN shifting costs β=.04, p=.66; parent age of onset: AGN inhibitory control errors β=.02, p=.74, VF mental generativity β=.01, p=.90, AGN shifting costs β=.03, p=.72; child exposure to previous parent depressive episodes: AGN inhibitory control errors β=−.10, p=.17, VF mental generativity β=.11, p=.08, AGN shifting costs β=.04, p=.61] or with offspring depressive symptoms [parent previous severe episode β=.06, p=.36; parent age of onset β=.05, p=.42; child exposure to previous parent depressive episodes β=.004, p=.95].

### 3.3 Buffering effect of executive functions on the association between current parental depression and adolescent total depressive symptoms

We next tested whether executive functioning moderated the association between current parental depression and adolescent depressive symptoms. We tested this separately for each executive functioning measure while controlling for covariates associated with offspring executive functioning or offspring depressive symptoms (age, gender and IQ, Table 4; Step 4). Results were consistent across the three measures of executive functioning which assess aspects of inhibitory control and mental flexibility.

#### 3.3.1 Main effects

In all three analyses lower adolescent IQ was associated with a greater number of adolescent depressive symptoms [AGN Inhibitory control errors: β=−.15, p<.05; VF mental generativity: β=−.13, p=.06; AGN shifting costs: β=−.16 p<.05]. Consistent with previous research and as expected, parental current depression was also associated with adolescent depressive symptoms. Thus, the number of depressive symptoms was significantly higher in adolescents whose parents met DSM-IV criteria for a current depressive episode in all three analyses [AGN Inhibitory control errors: β=.33, p<.001; VF mental generativity: β=.20, p<.01; AGN Shifting costs: β=.30 p<.001]. There were no significant main effects of executive functioning on adolescent depressive symptoms although a trend was observed for inhibitory control [AGN Inhibitory control errors: β=.15, p=.052; VF mental generativity: β=.02, p=.75; AGN Shifting costs: β=−.09 p=.23].

#### 3.3.2 Interactive effects

**Fig. 1:**
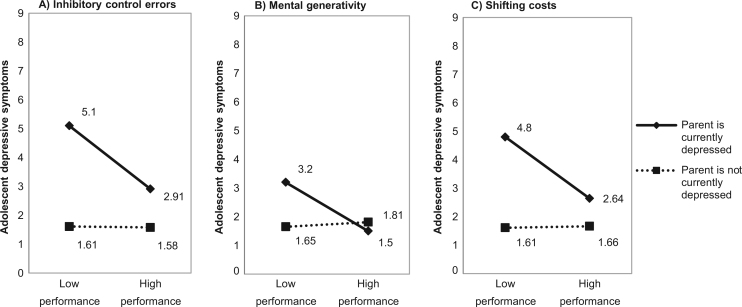
The interaction between current parental depression and measures of executive functioning as a predictor of adolescent depressive symptoms.

Results of the interaction terms between current parental depression and executive functioning measures showed that both AGN inhibitory control errors and AGN shifting costs significantly moderated the association between current parental depression and offspring depressive symptoms in the expected direction [AGN Inhibitory control errors: β=.16, p<.05; AGN Shifting costs: β=.18, p<.05], where more errors of inhibitory control and greater shifting costs were associated with increased adolescent depressive symptoms. The interaction between current parental depression and mental generativity was associated at trend level with adolescent symptoms in the expected direction [VF mental generativity: β=−.13, p=.06] where lower mental generativity was associated with increased adolescent depressive symptoms. Results are presented graphically in order to illustrate the difference in depressive symptoms for adolescents with higher and lower performance on executive functioning measures, separately for those exposed and not exposed to a current parental depressive episode (Fig. 1). High and low performance in EF measures were defined as scoring in the top or bottom tertile of the distribution, respectively. On average, higher executive functioning was associated with a decrease of two depressive symptoms in the presence of a current parental episode. The same pattern of interactive effects between current parental depression and executive functioning measures emerged when analyses were conducted with adolescent-rated depressive symptoms as the outcome [AGN Inhibitory control errors: β=.15, p<.05; VF mental generativity: β=−.12, p=.08; AGN Shifting costs: β=.19, p<.05]. Interactions also replicated when excluding adolescents with a diagnosis of ADHD and were significant for all three measures of executive functioning [Combined parent-adolescent depressive symptoms: AGN Inhibitory control errors β=.19, p<.05; VF mental generativity β=−.18, p<.01; AGN Shifting costs β=.21, p<.01; Adolescent-rated depressive symptoms: AGN Inhibitory control errors β=.20, p<.05; VF mental generativity β=−.16, p<.05; AGN Shifting costs β=.21, p<.05]. Furthermore, when we repeated the analyses while controlling for the three additional parental depression variables (parent previous severe episode, parent age of onset and child exposure to previous parent depressive episodes), none of the additional parent depression covariates were associated with the outcome measure, and the general pattern of interactive effects replicates with only minor differences (Table S1).

### 3.4 Adolescent Cognitive-emotional depressive symptoms and vegetative-somatic depressive symptoms

We next examined whether the buffering effect of executive functioning measures was present for both cognitive-emotional symptoms and vegetative-somatic symptoms. The interaction between all three executive function measures and current parental depression was consistently associated with adolescent cognitive-emotional depressive symptoms in the expected direction [AGN Inhibitory control errors: β=.14, p<.05; VF mental generativity: β=−.17, p<.05; AGN Shifting costs: β=.21, p<.01; Table 5]. Only the interaction between current parental depression and AGN inhibitory control errors was significantly associated with vegetative-somatic symptoms (β=.15, p<.05). The interactions for the other two measures of executive functioning were non–significant [VF mental generativity: β=−.07, p=.32; AGN shifting costs β=.12, p=.14].

## 4 Discussion

This is the first study to examine whether higher executive functions confer protection against adolescent depressive symptoms in the presence of a current episode of parental depression. Consistent with a number of previous studies and a previous analysis of this cohort (Mars et al., 2012), we confirmed that within a high-risk cohort of adolescents, a current episode of MDD in the parent was associated with higher levels of depressive symptoms in the offspring. Our main findings indicated that when a parent was in a current episode of depression, adolescents with better executive functioning had significantly fewer total depressive symptoms compared to adolescents with poorer performance on these measures. Previous research has emphasized the importance of executive functioning in regulating thoughts and emotions and in coping with stress (Sanchez et al., 2013). Mental flexibility has been suggested to be associated with mechanisms underlying effective coping with adversity such as the generation of solutions to problems and the positive reappraisal of negative events (Diamond, 2013, McRae et al., 2012, Stahl and Pry, 2005). Although further research is required to examine the mechanisms behind the buffering effects of executive functioning observed in this study, our findings suggest that inhibitory control and mental flexibility may serve as important cognitive resources that facilitate the ability of young people to cope with having a currently depressed parent.

The mean difference in DSM-IV total depressive symptoms between adolescents with higher (upper tertile) versus lower (lower tertile) executive functioning was fairly substantial, around two depressive symptoms that met strictly defined thresholds according to a semi-structured clinical interview. As the DSM-IV diagnostic criteria for a major depressive episode requires the presence of 5 symptoms (out of 9 possible symptoms) this difference might have clinical and functional implications and highlights the importance of executive functioning as a potential protective factor. Thus, executive functioning appears to be a potentially clinically important protective factor for the adolescent offspring of depressed parents. Whilst interventions targeting executive functioning have been assessed for educational and neurodevelopmental difficulties in children and adults as well as for currently depressed adults (Melby-Lervåg and Hulme, 2013, Rapport et al., 2013, Siegle et al., 2007, Titz and Karbach, 2014), to our knowledge such programmes have not been tested in relation to individuals at high risk for mood disorders. It has been suggested that common intervention strategies such as CBT, encourage patients to employ “executive control” over negative automatic thoughts and affective responses (Siegle et al., 2007). It is possible that those with difficulties in executive functioning find these requirements of CBT demanding and considering enhancing executive functioning as an adjunct to more traditional preventive approaches may be warranted.

It is worth noting that inhibitory control and shifting costs were assessed by a task that involves the processing of affective information whereas mental generativity was assessed by a task that did not involve an explicit emotional component. Previous research has emphasized cognitive control impairments in the context of emotionally salient information as a factor associated with risk for depression and persistence of symptoms (Gotlib and Joormann, 2010, Gotlib et al., 2014, Kilford et al., 2014) which suggests that the buffering effect observed in the present study might be expected to be larger for ‘emotional’ executive functioning. Overall, the present study found relatively little evidence of this with similar effect sizes observed for both types of executive functioning task. Although the buffering effect of VF mental generativity only approached significance we did not interpret it differently from the significant buffering effects of AGN inhibitory control errors and AGN shifting costs as each of the three interactive effects had similar effect sizes and we did not directly test whether effects sizes were significantly different. We also found little evidence of valence specific effects when the interaction between current parental depression and AGN inhibitory control errors was examined separately for inhibitory control errors for blocks with a negative target and blocks with a positive target [negative target blocks (β=.17, p<.05); positive target blocks (β=.15, p<.05)]. There was some indication that the protective effects of executive functioning were somewhat more consistently observed for cognitive-emotional symptoms than for vegetative-somatic symptoms. However, it is important to note that we did not formally test differences. Executive functioning might be predicted to particularly confer protection against cognitive-emotional depressive symptoms given the important role that executive functions have in emotional regulation. The finding that only the interaction with inhibitory control and current parent depression was significant for both cognitive-emotional and vegetative-somatic symptoms is intriguing but requires replication.

We did not find evidence for significant main effects of executive functioning measures on adolescent depressive symptoms and instead found a main effect of IQ on adolescent depressive symptoms. Possible reasons for no main effect of EF on adolescent depressive symptoms include differences between depressive symptoms and disorder (Wagner et al., 2014) and that EF impairments may be related to current and past severity and/or chronicity in those with MDD (Basso and Bornstein, 1999, Karabekiroğlu et al., 2010, McDermott and Ebmeier, 2009). The finding of a main effect for IQ on adolescent depressive symptoms is consistent with evidence that IQ protects against familial risk for depression and other psychiatric disorders (Kendler et al., 2015, Zammit et al., 2004). It is also worth noting that overall findings in this study are consistent with findings in a previous examination of the associations between measures of parental depression severity and course and adolescent depressive symptoms in this cohort (Mars et al., 2012). Minor differences in results are related to the use of different analyses and different outcome measures. We did not find evidence for main effects of current parental depression as well as other indicators of parental depression (e.g. age of onset, past severity and child exposure to previous parent depressive episodes) on adolescent executive functioning. A few studies have examined whether there is an association between parental depression and offspring executive functions and results have been mixed. Hughes et al. (2013), found that mothers’ depressive symptoms when the child age was 2 as well as the change in maternal depressive symptoms between the child ages of 2-6, predicted childrens' executive functioning at age 6 in a community sample of low income families. Studies examining parental diagnosis of MDD have found no evidence of an association between parental depression and offspring EF (Klimes-Dougan et al., 2006, Micco et al., 2009, Wagner et al., 2015). This cross-sectional examination assessed adolescents whose parents had a history of recurrent depression, so it is not possible to rule out that other indicators of parental depression had earlier effects on offspring executive functions or that changes in parental depression had effects on the development of offspring EF over time.

We conducted analyses for three executive functioning measures and as suggested by Rothman, 1990, Rothman, 2014 did not correct for multiple comparisons. We examined comorbidity with ADHD but were not able to examine the role of comorbid anxiety because of the high correlation with depression in this sample (Gorman, 1996). It is therefore unclear whether anxiety may affect the observed buffering effect of EF on depressive symptoms. Further research is required in order to investigate this. Additional limitations include the cross-sectional and observational nature of the data. Thus, it is important to note that both unmeasured environmental and genetic factors, that is, residual confounding, could account for the associations between parent depression and offspring depression and between offspring executive functioning and offspring depression. As we have conducted a cross sectional examination of the association between a current episode in the parent and offspring depressive symptoms we are not able to infer any causal relationship between indicators of parental depression and offspring depressive symptoms. Whilst it is possible that reduced EF may stem from aspects of depression not examined in this study such as rumination (Davis and Nolen-Hoeksema, 2000, Watkins and Brown, 2002) or from motivational problems (Ellis, 1991), if poor EF was a result of depressed mood or another feature of depression such as rumination, a significant correlation between depressive symptoms and EF performance measures would be expected. In this sample we did not find such correlations (Table 1; results available from first author) but rather an interaction effect of parental depression and EF measures on depressive symptoms. Findings of a protective effect of executive functioning are cross-sectional meaning that the protective effect on depressive symptomatology may not persist over time. The focus on a high-risk sample of adolescents may also limit the extent to which findings generalise to other populations.

Further research is required to elucidate the pathways through which risk for depression is transmitted from parents to offspring and examine how executive functions may buffer this risk. Thus, longitudinal studies that assess EF early in life and prior to the development of offspring depression are warranted in order to rule out the possibility of earlier effects of parental or offspring depression on the development of offspring executive functions. It would also be informative to examine EF as a mediator of depressive symptom change in preventive trials of high risk groups and to consider the possibility of incorporating EF training in prevention programs aimed at increasing resilience in these groups.

## 5 Conclusion

This is the first study to demonstrate that executive functions may protect against depressive symptoms when adolescents are exposed to a current episode of parental depression. The assessment of executive function skills may help in the early detection of vulnerable individuals that are likely to be less ‘resilient’ when exposed to parental depression. These findings have therapeutic implications as preventive interventions could target executive functions as a way to promote resilience and to enhance the efficacy of existing interventions in high risk groups.

## Figures and Tables

**Table 1:** Descriptive statistics and correlations between study variables.

	Mean (SD) or N%	Range (Min, Max)	1	2	3	4	5	6	7	8	9	10	11	12	13
1. Age of child	13.77 (2.04)	8 (10,18)													
2. Gender of child (%male, % female)	40.3, 59.7	–	.08												
3. IQ of child	96.02 (12.67)	81 (50,131)	−.15⁎	.11											
4. Current parental depression (%yes, %no)	18.2, 81.8	–	−.05	.02	−.06										
5. Parental age of onset (%≤20,%>20)	27, 73	–	−.002	−.08	−.04	−.15⁎									
6. Parent previous severe episode (% yes, % no)	27.8, 72.2	–	.06	−.06	.02	.26⁎⁎⁎	−.22⁎⁎								
7. Child exposure to previous parent depressive episodes	3.8 (12.8)	170 (0,170)	.04	−.03	.02	.01	−.06	−.02							
8. Mental generativity (VF task)	27.14 (8.58)	46 (8,54)	.22⁎⁎⁎	.06	.40⁎⁎⁎	−.08	.02	.02	.09						
9. Inhibitory control errors (AGN task)	19.52 (13.26)	66 (1,67)	−.37⁎⁎	−.18⁎	−.12	.02	.10	.02	−.13	−.18⁎					
10. Shifting costs (AGN task; msecs)	−11.65 (44.04)	320.72 (−200.52,120.20)	.06	.08	−.11	.02	.04	.06	.04	−.03	−.05				
11. Adolescent total depressive SY	1.93 (1.94)	9 (0,9)	.19⁎⁎	.12⁎	−.16⁎⁎	.14⁎	−.01	.10	.01	−.03	.06	.06			
12. Adolescent cognitive- emotional SY	.65 (1.08)	5 (0,5)	.10	.15⁎	−.19⁎⁎	.18⁎⁎	−.02	.16⁎⁎	−.04	−.06	.04	.05	.86⁎⁎⁎		
13. Adolescent vegetative- somatic SY	1.27 (1.13)	4 (0,4)	.22⁎⁎⁎	.09	−.09	.08	−.04	.03	.05	.01	.07	.05	.88⁎⁎⁎	.53⁎⁎⁎	

SY= Symptoms;Min=minimal value; Max=maximal value; msec=milliseconds.

Correlations were calculated for 288 adolescents for whom psychopathology data was available; N ranges from 183 to 288 due to missing values.

⁎ p<.05.

⁎⁎ p<.01.

⁎⁎⁎ p<.001.

**Table 2:** Exploratory factor analysis on measures of executive functioning from the AGN and VF tasks.

**Measure**	**Descriptive data (N=186)**		**Factor Loadings**			
	Mean (SD)	Range	Attention	Inhibitory control errors	Mental Generativity	Shifting costs
AGN: omissions (shift blocks)	8.40 (6.78)	33	**.99**	−.07	.06	−.05
AGN: Total omissions	16.56 (13.02)	66	**.99**	−.05	.05	−.05
AGN: commissions (shift blocks)	10.30 (6.75)	36	−.04	**.98**	.04	−.05
AGN: Total commissions	19.62 (13.23)	66	−.06	**.98**	.04	−.04
VF: repetitions	.42 (1.12)	11	.14	.11	**.95**	.10
VF: correct responses	27.06 (8.37)	42	−.39	−.28	**.47**	−.20
AGN: shifting costs (msecs)	-11.65 (44.04)	320.72	−.09	.03	.06	**.75**
VF: disallowed responses	.99 (1.78)	12	−.03	.10	.01	**−.69**

**Factor**			**Correlations among factors**			
Attention						
Inhibitory control errors			.05			
Mental generativity			−.14	−.09		
Shifting costs			.06	−.05	−.07	

The Kaiser-Meyer-Olkin measure verified the sampling adequacy for the analysis, KMO=.50. Bartlett's test of sphericity χ^2^ (28)=1070.45, p<.001 indicated that correlations between measures were sufficiently large for exploratory factor analysis; Loadings of .4 or more were considered informative.

Descriptive data is presented for subjects with data on all measures of AGN and VF tasks. Minor variations in the descriptive data presented in Table 1, Table 2 are due to the use of pairwise deletion in Table 1 and listwise deletion in Table 2.

**Table 3:** Main effects of parent current depression, parent past severity, parent age of onset and child exposure to previous parent depressive episodes on offspring executive functioning and depressive symptoms.

	**Inhibitory control errors (N=172)**					**Mental generativity (N=239)**					**Shifting costs (N=171)**					**Adolescent depressive symptoms (N=257)**				
	**∆ R^2^**	**p**	**B (S.E.)**	**β**	**p**	**∆ R^2^**	**p**	**B (S.E.)**	**β**	**p**	**∆ R^2^**	**p**	**B (S.E.)**	**Β**	**P**	**∆ R^2^**	**p**	**B (S.E.)**	**β**	**p**
**Step 1**	.18	<.001				.04	<.01				.01	.39				.04	<.01			
Gender of child			−4.69(1.89)	−.17	<.05			.74(1.12)	.04	.51			5.46(6.84)	.06	.43			.37(.24)	.09	.13
Age of child			−5.16(.96)	−.37	<.001			1.80(.56)	.20	<.01			3.86(3.49)	.08	.27			.33(.12)	.17	<.01
**Step 2**	.001	.70				.01	.13				.002	.61				.04	<.01			
Gender of child			−4.67(1.90)	−.17	<.05			.75(1.12)	.04	.50			5.27(6.86)	.06	.44			.35(.24)	.09	.14
Age of child			−5.18(.97)	−.38	<.001			1.77(.56)	.20	<.01			3.93(3.50)	.09	.26			.35(.12)	.18	<.01
Current PD			−.95(2.44)	−.03	.70			−2.18(1.45)	−.10	.13			4.57(8.91)	.04	.61			1.03(.31)	.20	<.01
**Step 3**	.01	.53				.02	.30				.003	.93				.005	.73			
Gender of child			−4.51(1.91)	−.17	<.05			.79(1.13)	.04	.48			5.48(6.95)	.06	.43			.38(.24)	.10	.12
Age of child			−5.14(.98)	−.37	<.001			1.74(.56)	.20	<.01			3.64(3.55)	.08	.31			.34(.12)	.18	<.01
Current PD			−1.12(2.57)	−.03	.66			−2.34(1.50)	−.10	.12			4.28(9.41)	.04	.65			.99(.32)	.20	<.01
Parent previous severe episode			.41(2.25)	.01	.86			1.10(1.31)	.06	.40			3.56(8.18)	.04	.66			.25(.27)	.06	.36
Parent age of onset			.75(2.28)	.02	.74			.16(1.32)	.01	.90			2.96(8.29)	.03	.72			.22(.27)	.05	.42
Child exposure to previous parent depressive episodes			−4.99(3.66)	−.10	.17			4.26(2.41)	.11	.08			6.68(13.24)	.04	.61			.01(.13)	.004	.95

PD=Parental depression.

Total R^2^ of regression analyses: Inhibitory control errors: Total R^2^=.19; mental generativity: Total R^2^=.07; shifting costs: Total R^2^=.02; Adolescent depressive symptoms: Total R^2^=.09.

**Table 4:** Current parental depression, offspring executive functioning and their interaction predicting offspring depressive symptom count.

	**AGN Inhibitory control errors (N=182)**					**VF Mental generativity (N=254)**					**AGN Shifting costs (N=181)**				
	**∆ R^2^**	**p**	**B (S.E.)**	**β**	**p**	**∆ R^2^**	**p**	**B (S.E.)**	**β**	**p**	**∆ R^2^**	**p**	**B (S.E.)**	**β**	**p**
**Step 1**	.05	<.05				.06	<.001				.05	<.05			
Gender of child			.43(.29)	.11	.13			.48(.24)	.12	<.05			.42(.29)	.11	.15
Age of child			.37(.15)	.18	<.05			.40(.12)	.20	<.01			.36(.15)	.18	<.05
**Step 2**	.04	<.01				.03	<.01				.04	<.01			
Gender of child			.55(.29)	.14	.06			.56(.24)	.14	<.05			.54(.29)	.14	.06
Age of child			.32(.14)	.16	<.05			.36(.12)	.18	<.01			.31(.14)	.16	<.05
IQ of child			-.44(.16)	−.20	<.01			−.34(.13)	−.17	<.01			−.43(.16)	−.20	<.01
**Step 3**	.13	<.001				.04	<.01				.10	<.001			
Gender of child			.65(.27)	.16	<.05			.53(.23)	.14	<.05			.45(.28)	.12	.10
Age of child			.53(.14)	.27	<.001			.39(.12)	.20	<.01			.36(.14)	.18	<.05
IQ of child			−.33(.15)	−.15	<.05			−.29(.14)	−.14	<.05			−.38(.15)	−.18	<.05
Current PD			1.59(.34)	.32	<.001			1.04(.29)	.21	<.01			1.59(.35)	.32	<.001
EF measure (child)			.44(.15)	.22	<.01			−.06(.13)	−.03	.65			−.02(.14)	−.01	.86
**Step 4**	.02	<.05				.01	.06				.02	<.05			
Gender of child			.58(.27)	.15	<.05			.50(.23)	.13	<.05			.36(.27)	.09	.19
Age of child			.51(.14)	.26	<.01			.38(.12)	.20	<.01			.38(.14)	.19	<.01
IQ of child			−.32(.15)	−.15	<.05			−.26(.14)	−.13	.06			−.34(.15)	−.16	<.05
Current PD			1.63 (.33)	.33	<.001			.99(.29)	.20	<.01			1.54(.34)	.30	<.001
EF measure (child)			.31 (.16)	.15	.05			.04(.14)	.02	.75			−.18(.15)	−.09	.23
EF measure (child) X current PD			.92(.40)	.16	<.05			−.54(.28)	−.13	.06			.78(.33)	.18	<.05

PD= Parental depression.

Total R^2^ of regression analyses: AGN Inhibitory control errors: R^2^=.24; VF Mental generativity: R^2^=.15; AGN Shifting costs: R^2^=.21.

Coding of the variables: AGN Inhibitory control errors: higher scores represent worse performance; VF Mental generativity: higher scores represent better performance; AGN Shifting costs: higher scores represent worse performance.

**Table 5:** Current parental depression, offspring executive functioning and their interaction predicting offspring cognitive-emotional and vegetative-somatic depressive symptoms.

	**Inhibitory control errors**										**Mental generativity**										**Shifting costs**									
	**Cognitive symptoms (N=183)**					**Vegetative symptoms (N=184)**					**Cognitive symptoms (N=256)**					**Vegetative symptoms (N=258)**					**Cognitive symptoms (N=182)**					**Vegetative symptoms (N=183)**				
	**∆R^2^**	**p**	**B(S.E.)**	**β**	**p**	**∆R^2^**	**p**	**B(S.E.)**	**β**	**p**	**∆R^2^**	**p**	**B(S.E.)**	**β**	**p**	**∆R^2^**	**p**	**B(S.E.)**	**β**	**p**	**∆R^2^**	**p**	**B(S.E.)**	**β**	**p**	**∆R^2^**	**p**	**B(S.E.)**	**β**	**p**
**Step 1**	.02	.17				.07	<.01				.02	.07				.08	<.001				.02	.19				.06	<.01			
Gender of child			.25(.16)	.12	.12			.20(.17)	.09	.23			.22(.13)	.10	.09			.22(.14)	.10	.11			.24(.16)	.11	.127			.19(.17)	.08	.26
Age of child			.08(.08)	.07	.33			.28(.08)	.24	<.01			.10(.07)	.09	.14			.29(.07)	.25	<.001			.08(.08)	.07	.351			.28(.08)	.24	<.01
**Step 2**	.05	<.01				.02	<.05				.03	<.01				.01	.11				.04	<.01				.02	.05			
Gender of child			.31(.16)	.15	<.05			.25(.17)	.11	.14			.27(.13)	.13	<.05			.25(.14)	.11	.07			.31(.16)	.14	.051			.24(.17)	.10	.16
Age of child			.05(.08)	.05	.52			.26(.08)	.22	<.01			.08(.07)	.07	.25			.28(.07)	.24	<.001			.05(.08)	.04	.537			.26(.08)	.22	<.01
IQ of child			−.26(.09)	−.22	<.01			−.18(.09)	−.14	<.05			−.21(.07)	−.18	<.01			−.12(.07)	−.10	.11			−.25(.09)	−.22	<.01			−.18(.09)	-.14	.05
**Step 3**	.12	<.001				.09	<.001				.06	<.001				.02	.10				.11	<.001				.05	<.01			
Gender of child			.32(.15)	.15	<.05			.33(.16)	.14	<.05			.25(.13)	.12	.05			.24(.14)	.10	.09			.26(.15)	.12	.083			.21(.16)	.09	.21
Age of child			.14(.08)	.13	.09			.38(.09)	.33	<.001			.09(.07)	.09	.17			.29(.07)	.25	<.001			.08(.08)	.07	.300			.28(.08)	.24	<.01
IQ of child			−.21(.08)	−.18	<.05			−.12(.09)	-.10	.18			−.19(.08)	−.16	<.05			−.10(.08)	−.08	.25			−.22(.08)	-.19	<.01			−.16(.9)	−.12	.08
Current PD			.92(.18)	.34	<.001			.66(.20)	.22	<.01			.65(.16)	.24	<.001			.37(.18)	.13	<.05			.92(.19)	.34	<.001			.66(.21)	.22	<.01
EF measure (child)			.15(.08)	.14	.07			.28(.09)	.24	<.01			−.02(.07)	−.02	.81			−.03(.08)	−.03	.68			−.01(.07)	−.01	.873			−.01(.08)	−.01	.87
**Step 4**	.02	<.05				.02	<.05				.02	<.05				.004	.32				.03	<.01				.01	.14			
Gender of child			.29(.15)	.14	.05			.29(.16)	.12	.08			.24(.13)	.11	.07			.23(.14)	.10	.10			.20(.15)	.10	.171			.17(.17)	.07	.30
Age of child			.12(.08)	.12	.12			.37(.09)	.32	<.001			.08(.07)	.08	.20			.29(.07)	.25	<.001			.09(.07)	.08	.233			.28(.08)	.24	<.01
IQ of child			−.20(.08)	−.17	<.05			−.12(.09)	-.09	.19			−.16(.08)	−.14	<.05			−.09(.08)	−.07	.30			−.20(.08)	−.17	<.05			−.14(.09)	−.11	.12
Current PD			.94(.18)	.35	<.001			.69(.20)	.23	<.01			.61(.16)	.22	<.001			.36(.18)	.12	<.05			.89(.19)	.33	<.001			.64(.21)	.22	<.01
EF measure (child)			.08(.09)	.08	.33			.21(.09)	.18	<.05			.06(.08)	.06	.45			.00(.08)	.00	.99			−.11(.08)	−.10	.189			−.07(.09)	−.06	.43
EF measure (child) X Current PD			.44(.22)	.14	<.05			.49(.24)	.15	<.05			−.40(.16)	−.17	<.05			−.17(.17)	−.07	.32			.48(.18)	.21	<.01			.30(.20)	.12	.14

PD=Parental depression.

Total R^2^ of regression analyses: Inhibitory control errors: Cognitive symptoms- R^2^=.21; Vegetative symptoms- R^2^=.20, Mental generativity: Cognitive symptoms- R^2^=.13; Vegetative symptoms- R^2^=.11; Shifting costs: Cognitive symptoms – R^2^=.21; Vegetative symptoms – R^2^=.14.
