# Supplementary material for: Do better executive functions buffer the effect of current parental depression on adolescent depressive symptoms?
Source: J Affect Disord. 2016 Jul 15;199:54–64. doi: 10.1016/j.jad.2016.03.049 (PMC4871808; doi:10.1016/j.jad.2016.03.049)
Supplement: Supplementary file 1 — Supplementary material [file mmc1.docx]

**Supplementary material**

**Figure S1.** Participation rates and reasons for non-completion of executive functioning tasks

**Psychiatric interviews conducted with:**

288 parents

275 adolescents

N=288 (parents or adolescents)

N= 288 parents or children

**neuro-cognitive battery conducted with:**

264* adolescents

187 completed AGN

**Non completion AGN**

**n=76**

(Technical problems or equipment shortage (n=45); time constraints (n=13); refusal (n=6); child not at home (n=1); other e.g. injured (n=3); reason not specified (n=8))

**VF task conducted with:**

264 adolescents

**Non completion VF**

**n=24**

(time constraints (n=1); refusal (n=6); child not at home (n=4); other (n=5); reason not specified (n=8))

*One of these subjects only completed the verbal fluency task and was unable to complete the battery in full due to time constraints.
