# Supplementary material for: Do better executive functions buffer the effect of current parental depression on adolescent depressive symptoms?
Source: J Affect Disord. 2016 Jul 15;199:54–64. doi: 10.1016/j.jad.2016.03.049 (PMC4871808; doi:10.1016/j.jad.2016.03.049)
Supplement: Supplementary file 2 — Supplementary material [file mmc2.docx]

**Supplementary material**

**Table S1.** Current parental depression, offspring executive functioning and their interaction predicting offspring depressive symptom count while controlling for additional parental depression variables.

|  | **AGN Inhibitory control errors (N=167)** | | | **VF Mental generativity (N=231)** | | | **AGN Shifting costs (N=166)** | | |
| --- | --- | --- | --- | --- | --- | --- | --- | --- | --- |
|  | **B(S.E.)** | **β** | **p** | **B(S.E.)** | **β** | **p** | **B(S.E.)** | **β** | **p** |
| Gender of child | .51 (.29) | .13 | .08 | .48 (.25) | .12 | .06 | .33 (.29) | .08 | .27 |
| Age of child | .45 (.16) | .22 | <.01 | .34 (.13) | .17 | <.05 | .29 (.15) | .14 | .06 |
| IQ of child | -.32 (.16) | -.15 | <.05 | -.25 (.15) | -.12 | .09 | -.34 (.16) | -.16 | <.05 |
| Current PD | 1.72 (.38) | .33 | <.001 | .98 (.33) | .19 | <.01 | 1.72 (.39) | .33 | <.001 |
| EF measure (child) | .30 (.17) | .15 | .07 | .04 (.15) | .02 | .77 | -.18 (.15) | -.09 | .23 |
| **Parent previous severe episode** | **.15 (.34)** | **.03** | **.65** | **.25 (.29)** | **.06** | **.39** | **.30 (.34)** | **.07** | **.37** |
| **Parent age of onset**  **onset** | **-.17 (.33)** | **-.04** | **.60** | **-.01 (.29)** | **-.002** | **.98** | **-.01 (.34)** | **-.002** | **.98** |
| **Child exposure to previous parent depressive episodes** | **.38 (.54)** | **.05** | **.47** | **.29 (.53)** | **.04** | **.59** | **.33 (.54)** | **.04** | **.54** |
| **EF measure (child) X current PD** | **.74 (.44)** | **.13** | **.09** | **-.68 (.31)** | **-.16** | **<.05** | **.91 (.38)** | **.18** | **<.05** |

The table presents the final regression model.

PD= Parental depression.

Total R² of regression analyses: AGN Inhibitory control errors: R²=.24; VF Mental generativity: R²=.15; AGN Shifting costs: R²=.22.
